# Supplementary material for: Transcriptome Analysis of Meloidogyne javanica and the Role of a C-Type Lectin in Parasitism
Source: Plants (Basel). 2024 Mar 4;13(5):730. doi: 10.3390/plants13050730 (PMC10935113; doi:10.3390/plants13050730)
Supplement: Supplementary file 1 [file plants-13-00730-s001.zip › plants-2851149-supplementary.pdf]

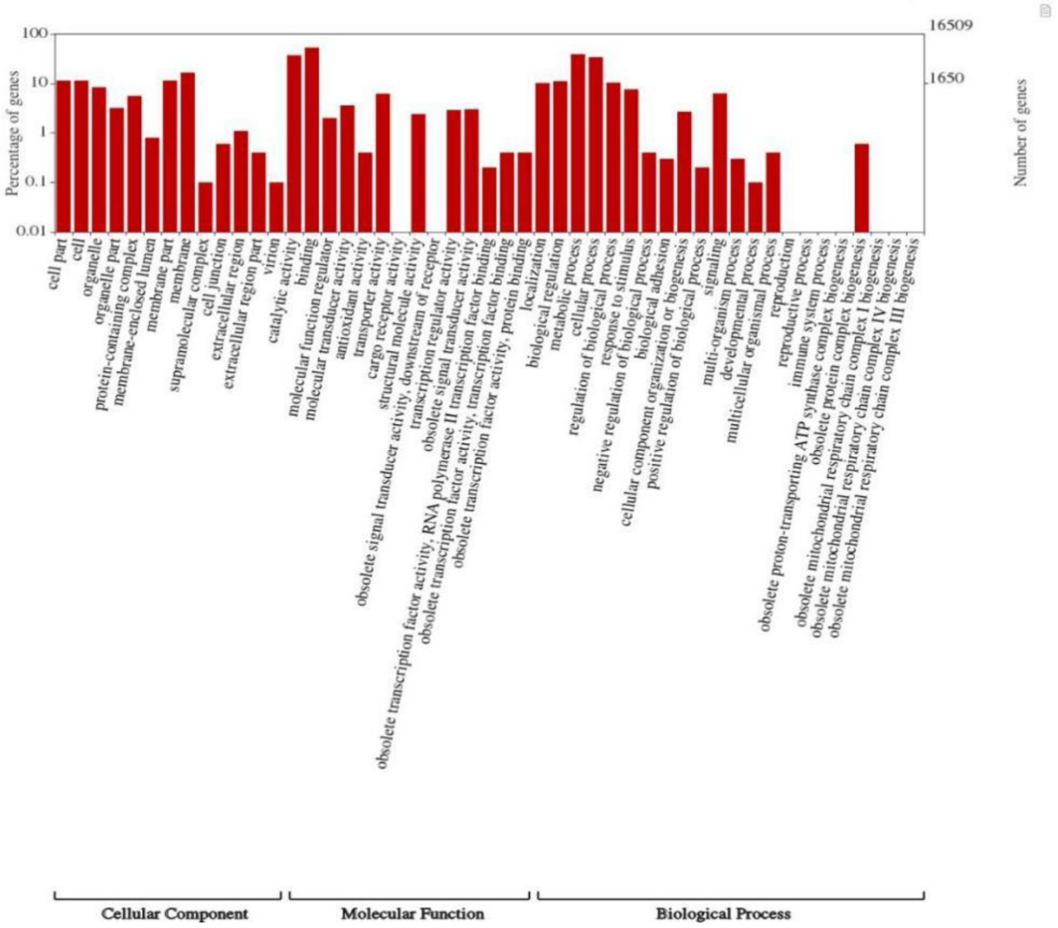

Figure S1 GO classification of unigenes in the transcriptome of *Meloidogyne javanica*. GO functions are shown on the X-axis. The right side of the Y-axis shows the number of genes with the GO function, and the left side shows the percentage.

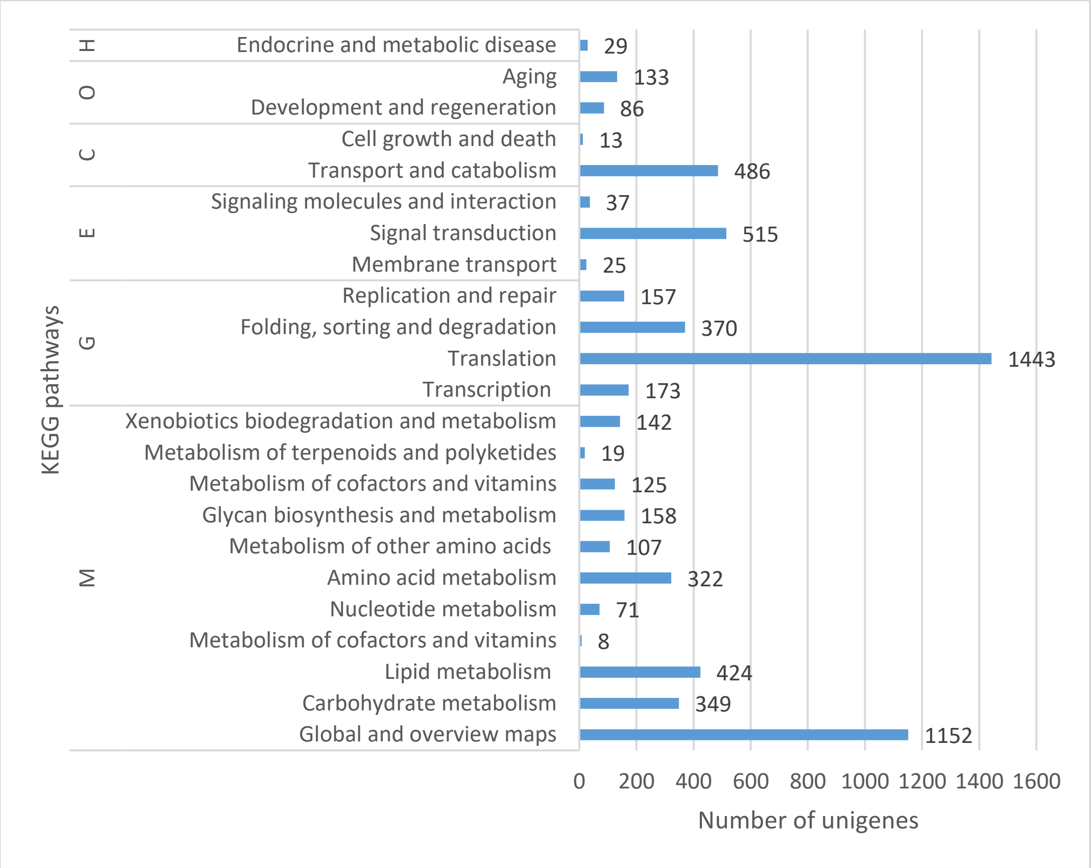

Figure S2 Distribution of the KEGG pathway of unigenes in the transcriptome of *Meloidogyne javanica*. KEGG pathways are shown on the Y-axis. The X-axis shows the number of genes with the KEGG pathway.M: Metabolism, G: Genetic Information Processing, E: Environmental Information Processing, C: Cellular Processes,O: Organismal Systems, H: Human Diseases

```
Majority MDPYKYRPSSAXNSPFXTTNSGAPVYNNNSSLTVGXRGPVLLEDYHLXEKLANFDRERIPERVVHARGASAKGFFEVTHD
                  10         20         30         40         50         60         70         80
1.pro    MDPSKYRPSSAYDTPFLTTNAGGPVYNNVSSLTVGPRGPVLLEDYYLIEKLATFDREKIPERVVHARGASAKGFFEVTHD 80
2.pro    MDPYKYRPSSAFNSPFCTTNSGAPVFNNNSSLTVGARGPVLLEDYHLVEKLANFDRERIAERVVHARGASAKGFFEVTHD 80
3.pro    MDPYKYRPSSAANSPFMTTNSGAPVYNNNSSLTVGTRGPILLEDYHLLEKIANFDRERIPERVVHARGASAKGFFEVTHD 80

Majority ISHLTCADFLRAPGVQTPVIVRFSTVIHERGSPETLRDPRGFAVKFYTREGNFDLVGNNFPVFFXRDGMKFPDMVHALKP
                  90        100        110        120        130        140        150        160
1.pro    ISHLTCADFLRAPGAQTPVICRFSTVVHERGSPESIRDIRGFAVKFYTREGNFDLVGNNVPVFFNRDAKSFPDTIRALKP 160
2.pro    IAHLTCADFLRAPGVQTPVIVRFSTVIHERGSPETLRDPRGFAVKFYTREGNFDLVGNNFPVFFIRDGMKFPDMVHALKP 160
3.pro    ISHLTCADFLRAPGVQTPVIVRFSTVIHERGSPETLRDPRGFAVKFYTREGNFDLVGNNFPVFFVRDGMKFPDMVHALKP 160

Majority NPKSHIQENWRILDFFSHHPESLHMFXFLFDDXGIPQDYRHMDGFGVHTYXLINKAGKAXYVKFHWKPTCGVKCLLEEEA
                 170        180        190        200        210        220        230        240
1.pro    NPKSHIQENWRILDFFSFLPESLHTFAFFYDDVCLPTDYRHMEGFGVHAYQLINKEGKAHYVKFHWKPTCGVKCMSEEEA 240
2.pro    NPKSHIQENWRVLDFFSHHPESLHMFTFLFDDIGIPQDYRHMDGSGVHTFTLINRAGKSTYVKFHWKPTCGVKSLLEEEA 240
3.pro    NPKSHIQENWRILDFFSHHPESLHMFSFLFDDLGIPQDYRHMDGFGVNTYMLINKAGKAQYVKFHWKPTCGVKCLLDEEA 240

Majority IRVGGXNHSHATKDLYDSIAAGNYPEWKLFIQTMDPEHEDKFDFDPLDVTKTWPEDILPLQPVGRLVLNRNIDNFFAENE
                 250        260        270        280        290        300        310        320
1.pro    IRVGGTNHSHATKDLYDSIAAGNYPEWKLFIQTMDPEDVDKFDFDPLDVTKTWPEDLLPLIPVGRLVLNRNIDNFFAENE 320
2.pro    IRVGGANHSHATQDLYDSIAAGNYPEWKLFIQIMDPEHEDKFDFDPLDVTKTWPEDILPLQPVGRLVLNKNIDNFFNENE 320
3.pro    IKVGGSNHSHATKDLYDSIAAGNYPEWKLFIQTIDPDHEDRFDFDPLDVTKIWPEDILPLQPVGRLVLNRNIDNFFAENE 320

Majority QLAFCPXIIVPGVYYSDDKLLQTRIFSYADTQRHRLGPNYLQLPVNAPKCAHHNNHXDGXMNFMHRDEEVDYLPSRXDPC
                 330        340        350        360        370        380        390        400
1.pro    QLAFNPGHIVPGIYYSEDKLLQTRIFAYADTQRHRIGPNYMQLPVNAPKCGHHNNHRDGAMNMTHRDEEVDYLPSRFDPC 400
2.pro    QLAFCPSIVVPGVYYSDDKMLQTRIFSYSDTQRYRLGPNYLQLPANAPKCAHHNNHYDGSMNFMHRDEEIDYFPSRYDQV 400
3.pro    QLAFCPAIIVPGVYYSDDKLLQTRVFSYADTQRHRLGPNYLQLPVNAPKCAHHNNHHEGLMNFMHRDEEVNYLPSRLDPC 400

Majority RHAEQYPIPSXVLXGKREKCIIXKENNFKQPGERYRSWXPDRQERFIXRWVEALSDPRXTHEIRSIWISYWSQADKSLGQ
                 410        420        430        440        450        460        470        480
1.pro    RPAEQYPIPSCVLNGRRTNCVIPKENNFKQAGERYRSWEPDRQDRYINKWVESLSDPRVTHEIRSIWISYLSQADKSCGQ 480
2.pro    RHAEVYPIPSTVCSGKREKCIIQKENNFKQPGERYRSFTPDRQERFIRRWVEALSDPRITYEIRSIWITYWSQADKSLGQ 480
3.pro    RHAEQYPIPPRVLTGKREKVIIEKENNFKQPGEHYRSWAPDRQERFLCRWVDALSDPRATHEIRSIWISYWSQADKSLGQ 480

Majority KLASRLNVRPTM-
                 490
1.pro    KVASRLTVKPTM.                                                                   493
2.pro    KLASRLNVRPSI.                                                                   493
3.pro    KLASRLNIRPTM.                                                                   493
```

Figure S3. Multiple sequence alignment of *Solanum lycopersicum* catalases.

Table S1 Summary of *M. javanica* transcriptomes sequencing

Sample	raw_reads	clean_reads	error_rate	Q20(%	Q30(%)	GC(%)
Par-J3/J4	65241136	55759032	0.03	96.89	91.9	37.94
Pre-J2	94398136	92886548	0.03	98.02	93.89	37.36

Table S2 The number and types of lectin genes on various plant parasitic nematodes. MG: *Meloidogyne graminicola*, HO: *Hirschmanniella oryzae*, HG: *Heterodera glycines*, PC: *Pratylenchus coffeae*, PT: *Pratylenchus thornei*, MJ: *Meloidogyne javanica*, #Seq: the number of protein sequences that was scanned for potential lectin domains.

Species	#Seq	Class V Chitinase	Legume	LysM	Ricin-B	Calreticulin	M-Type Lectin	C-Type Lectin	Galectin
MJ	48697	3	3	3	6	1	0	10	27
MG	10973	3	0	0	1	4	2	48	13
HO	17061	5	2	2	2	5	2	33	28
HA	10070	3	2	2	6	1	4	1	9
HG	12262	4	0	1	0	5	0	4	9
PC	11507	0	0	1	2	1	0	3	7
PT	19533	0	0	1	4	2	1	0	5

Table S3 The primers used in this paper.

Primer name	Sequence（5'-3'）	Application
Mj-ctl-2-ish-F	ATGTTCCCAAACTGGGCTTT	4.7
Mj-ctl-2-ish-R	CTGTGAATTGTAGCAACTTC	4.7
Mj-ctl-2-lq-F	ACTCTTCGCGTATTCGTCC	4.8
Mj-ctl-2-lq-R	CATTGGTCTGGCACTGGT	4.8
Mj-ctl-1-yxb-F	CACCAAATCGACTCTAGAATGATTGCTTTTTCTTCC	4.9
Mj-ctl-1-sp-yxb-F	CACCAAATCGACTCTAGACAAGCAACATGCCCTTCC	4.9
Mj-ctl-1-yxb-R	ACCATGGTGGCGGTACCTGGTTGATCACATTCTTT	4.9
Mj-ctl-2-yxb-F	AAA TCTAGA ATGATTTTCTTGTTATTTATAT	4.9
Mj-ctl-2-sp-yxb-F	AAA TCTAGA TTTGCTCCTTTCCATCCACC	4.9
Mj-ctl-2-yxb-R	AAA GGTACC TTTTTCTTCTTCATCATTATCA	4.9
Mj-ctl-3-yxb-F	AAA TCTAGA ATGCATCTTTCTTCTATTCT	4.9
Mj-ctl-3-sp-yxb-F	AAA TCTAGA CAACAAATACCGAATTGCCA	4.9
Mj-ctl-3-yxb-R	AAA GGTACC AACATCTTGAGCAGCACAGT	4.9
Mj-ctl-2-TRV-F	GGTACCCACGGTCATCACCCATTTCCA	4.11
Mj-ctl-2TRV-R	GGTACCAGAATATTCCAATGCGATGC	4.11

CAT1-AD-F	TGGCCATGGAGGCCAGTGAATTCATGGATCCCTCTAAGTATC	4.12
CAT1-AD-R	TCTGCAGCTCGAGCTCGGGATCCCATTGTAGGCTTCACAGTGA	4.12
CAT2-AD-F	TGGCCATGGAGGCCAGTGAATTCATGGATCCTTACAAGTACCG	4.12
CAT2-AD-R	TCTGCAGCTCGAGCTCGGGATCCTATGCTTGGTCTCACATTAA	4.12
CAT3-AD-F	TGGCCATGGAGGCCAGTGAATTCATGGATCCTTACAAGTATCG	4.12
CAT3-AD-R	TCTGCAGCTCGAGCTCGGGATCCCATTGTAGGCCTTATATTG	4.12
CTL-2-BD-F	CAGAGGAGGACCTGCATATGGCCATGATTTTCTTGTTATTTAT	4.12
CTL-2-BD-R	GCTGCAGGTCGACGGATCCTTTTTCTTCTTCATCATTATC	4.12
